# Effect of the Chemical Structure of Modifiers Used in the Receptive Membrane of an Umami Taste Sensor on Its Electrical Responses

**DOI:** 10.3390/s26061787

**Published:** 2026-03-12

**Authors:** Kiyoshi Toko, Sota Otsuka, Mariko Koshi, Yuzuki Koga, Takeshi Onodera, Rui Yatabe, Toshiro Matsui

**Affiliations:** 1 Food and Health Innovation Center, Nakamura Gakuen University, 5-7-1 Befu, Fukuoka 814-0198, Japan; 2 Graduate School of Nutritional Sciences, Nakamura Gakuen University, 5-7-1 Befu, Fukuoka 814-0198, Japan; 3 Research and Development Center for Five-Sense Devices, Kyushu University, 744 Motooka, Fukuoka 819-0395, Japan; tmatsui@agr.kyushu-u.ac.jp; 4 Institute for Advanced Study, Kyushu University, 744 Motooka, Fukuoka 819-0395, Japan; 5 Graduate School of Information Science and Electrical Engineering, Kyushu University, 744 Motooka, Fukuoka 819-0395, Japan; otsuka.sota.551@s.kyushu-u.ac.jp; 6 Department of Bioscience and Biotechnology, Faculty of Agriculture, Graduate School of Kyushu University, 744 Motooka, Nishi-ku, Fukuoka 819-0395, Japan; m.koshi@agr.kyushu-u.ac.jp (M.K.); y.koga@agr.kyushu-u.ac.jp (Y.K.); 7 Faculty of Information Science and Electrical Engineering, Kyushu University, 744 Motooka, Fukuoka 819-0395, Japan; onodera@ed.kyushu-u.ac.jp (T.O.); yatabe.rui.678@m.kyushu-u.ac.jp (R.Y.)

**Keywords:** taste sensor, umami, lipid/polymer membrane, modifiers, hydrogen bonding

## Abstract

In our previous study, a taste sensor employing a lipid/polymer membrane modified with 2,6-dihydroxyterephthalic acid (2,6-DHTPA) enabled the detection of the umami substances monosodium glutamate (MSG) and inosinate monophosphate (IMP). The taste sensor was also able to evaluate the synergistic effect, an umami enhancement phenomenon that occurs between MSG and IMP. However, the structural requirements for modifiers capable of detecting IMP have not yet been clarified. In the present study, to elucidate these requirements, nine different modifiers were prepared, and taste sensor measurements for IMP were conducted in combination with ^1^H-NMR analysis. As a result, three distinct patterns were observed: (1) modifiers that exhibited chemical shift changes and generated a potential response in the positive direction (i.e., a positive potential response); (2) modifiers that showed chemical shift changes but produced either an almost zero or a negative potential response; and (3) modifiers that exhibited neither chemical shift changes nor any potential response. For receptor membranes that did not exhibit a positive response, the corresponding modifiers either lacked two carboxyl groups or did not possess intramolecular hydrogen bonding involving hydroxyl groups. From these results, it was clarified that the essential conditions for obtaining a positive potential response to IMP are that the modifier (1) contains two carboxyl groups and (2) possesses intramolecular hydrogen bonding.

## 1. Introduction

Human taste perception is composed of five basic tastes: sweetness, umami, bitterness, sourness, and saltiness. In addition to these, pungency and astringency, which are physical sensations, are also perceived. These seven tastes are generally regarded as the major taste qualities. Umami was found approximately 110 years ago as the fifth basic taste [1,2]. Representative umami substances include the amino acid-derived monosodium glutamate (MSG), which is found in seaweeds, vegetables, legumes, and fermented foods, as well as the nucleotide-derived inosinate monophosphate (IMP), which is present in dried bonito, dried sardines, pork, and beef [1,2,3,4,5,6]. Umami substances not only enhance the flavor of foods and stimulate appetite but have also been reported to contribute to reducing the risks of hypertension and cardiovascular disease caused by excessive salt intake [7]. A synergistic effect, in which MSG and IMP mutually enhance umami intensity, is well known [5,8,9]. MSG is recognized by the T1R1/T1R3 receptor at the Venus flytrap domain near the hinge region, whereas IMP is known to bind near the entrance of the Venus flytrap domain [10,11,12]. In addition, metabotropic glutamate receptors such as mGluR1 and mGluR4 are also reported to respond to MSG [13].

Taste perception is inherently subjective. From the viewpoints of new product development and quality control, there has been a strong demand in the food and pharmaceutical industries for devices that can quantify and visualize taste. Conventional chemical analytical techniques cannot directly quantify or visualize human taste perception itself or evaluate interactions between taste substances or taste qualities, such as the synergistic effects described above or bitterness suppression, and they are not necessarily simple or rapid. In general, sensors are devices designed to detect and quantify target substances easily and quickly, and sensors specifically used to measure taste substances are referred to as electronic tongues or taste sensors. To date, taste sensors based on various principles and methodologies have been reported, including potentiometric, voltametric, and impedimetric types [14,15,16,17,18,19,20,21,22,23,24,25,26,27,28,29,30,31,32,33,34], sensors using biological tissues [35,36,37,38], and enzyme-based sensors [39,40].

Taste sensors utilizing lipid/polymer membranes have already been put into practical use worldwide and are capable of quantifying and visualizing the five basic tastes. However, with respect to umami measurement, the reception mechanism is still far from molecular recognition [41], resulting in insufficient selectivity for umami. In contrast with conventional chemical analytical methods, electronic tongues, or taste sensors, offer the major advantage of simultaneously measuring the five basic tastes and quantifying and visualizing food taste and quality. Therefore, it is highly desirable to achieve umami measurement within the framework of taste sensors.

Against this background, methods for modifying lipid/polymer membranes have been developed over the past couple of years, enabling the detection and quantification of the umami substance MSG [42,43,44]. Among the modifiers examined, 2-hydroxyterephthalic acid (2-HTPA) and 2,6-dihydroxyterephthalic acid (2,6-DHTPA) exhibited the largest potential response to MSG. These membranes generated a potential response in the positive direction (i.e., a positive potential response) upon exposure to MSG. Notably, they showed almost no response to other taste qualities such as sweetness and bitterness, indicating a high selectivity for umami [42,44]. At neutral pH, the carboxyl groups of, for example, 2,6-DHTPA are dissociated due to intramolecular hydrogen bonding with adjacent hydroxyl groups. In the presence of MSG, intermolecular hydrogen bonds are formed between MSG and the two carboxyl groups of 2,6-DHTPA. As a result, protons that had dissociated into the solution return to the carboxyl groups, rendering 2,6-DHTPA electrically neutral. Because MSG carries an approximate charge of −1, the membrane acquires a net positive charge relative to its initial charge of −2. This charge shift in the positive direction, caused by the transition from intramolecular to intermolecular hydrogen bonding, leads to a positive potential response of the sensor.

Based on this finding, the use of a 2,6-DHTPA-modified membrane enabled not only the detection of IMP but also the direct evaluation of the synergistic effect between MSG and IMP, as clearly demonstrated in [45]. However, because only a single modifier was employed, the detailed detection mechanism for IMP remained unclear, and the meaning of the positive potential response was not investigated in detail. Consequently, the structural requirements of modifiers necessary for IMP detection were not elucidated. The purpose of this study is to clarify it. Nine commercially available modifiers were selected to evaluate their interaction with IMP. We integrated taste sensor potentiometry with ^1^H-NMR spectroscopy, allowing us to correlate macroscopic electrochemical responses with microscopic molecular structures. Three response patterns emerged based on chemical shifts and potentials. A positive response specifically requires modifiers to possess both intramolecular hydrogen bonding and two carboxyl groups. Modifiers lacking these structural features failed to produce a positive potential, regardless of chemical shift changes.

## 2. Materials and Methods

### 2.1. Reagents

**Figure 1:**
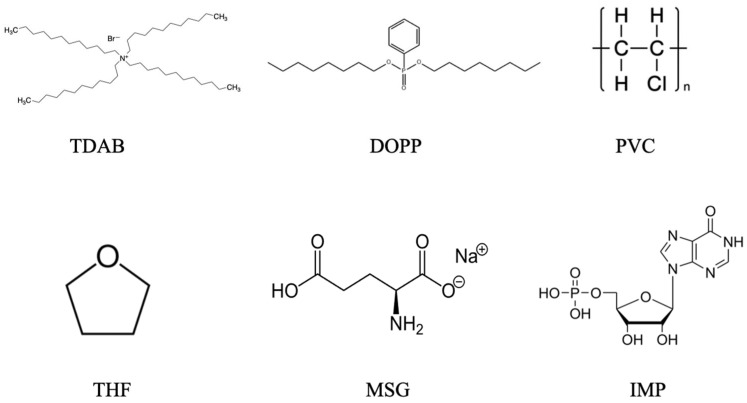
Chemical structures of TDAB, DOPP, PVC, THF, MSG, and IMP.

Tetradodecylammonium bromide (TDAB) lipids and tetrahydrofuran (THF) were obtained from Sigma-Aldrich (St. Louis, MO, USA). Dioctyl phenylphosphonate (DOPP), used as a plasticizer, was purchased from Dojindo Laboratories (Kumamoto, Japan). MSG, IMP, sodium chloride (NaCl), potassium chloride (KCl), and tartaric acid were supplied by Kanto Chemical Co., Inc. (Tokyo, Japan). Polyvinyl chloride (PVC) was obtained from FUJIFILM Wako Pure Chemical Corporation (Osaka, Japan). The molecular structures of TDAB, DOPP, PVC, and THF constituting the lipid/polymer membrane, together with those of the umami substances MSG and IMP used in the measurements, are illustrated in Figure 1.

**Figure 2:**
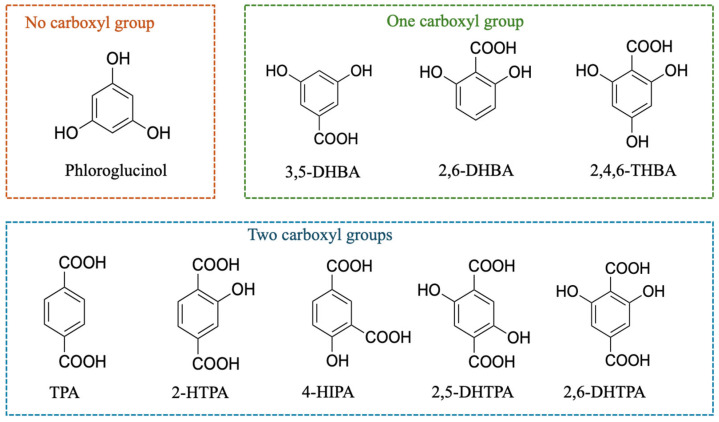
Chemical structures of nine modifiers: phloroglucinol, 3,5-DHBA, 2,6-DHBA, 2,4,6-THBA, TPA, 2-HTPA, 4-HIPA, 2,5-DHTPA, and 2,6-DHTPA.

Nine types of modifiers were employed in this study. 2,6-Dihydroxybenzoic acid (2,6-DHBA), 3,5-dihydroxybenzoic acid (3,5-DHBA), 2,4,6-trihydroxybenzoic acid (2,4,6-THBA), and phloroglucinol were purchased from FUJIFILM Wako Pure Chemical Corporation (Osaka, Japan). 2,6-DHTPA was obtained from BLDpharm (Shanghai, China). Terephthalic acid (TPA), 2-HTPA, 2,5-dihydroxyterephthalic acid (2,5-DHTPA), and 4-hydroxyisophthalic acid (4-HIPA) were supplied by Tokyo Chemical Industry (Tokyo, Japan). The chemical structures of these modifiers are shown in Figure 2.

As shown in Figure 2, phloroglucinol has no carboxyl groups, whereas 3,5-DHBA, 2,6-DHBA, and 2,4,6-THBA each possess one carboxyl group. In contrast, TPA, 2-HTPA, 4-HIPA, 2,5-DHTPA, and 2,6-DHTPA have two carboxyl groups. Among the modifiers containing carboxyl groups, 3,5-DHBA and TPA do not have adjacent hydroxyl groups and therefore do not form intramolecular hydrogen bonds. The other modifiers, such as 2,6-DHBA and 2-HTPA, which possess one or two carboxyl groups, are capable of forming intramolecular hydrogen bonds.

### 2.2. Preparation of the Lipid/Polymer Membrane

Sensor electrodes incorporating a lipid/polymer membrane were used for taste measurements. The membrane was prepared by dissolving TDAB (10 mM, 1 mL), DOPP (1.5 mL), and PVC (800 mg) in 9 mL of THF. The resulting solution was poured into a 90 mm-diameter Petri dish, and THF was allowed to evaporate to form the membrane. The membrane was further dried in a covered Petri dish at room temperature (25 °C) for 24 h. After drying, the membrane was cut into pieces of approximately 10 mm × 6 mm and used for measurements. The membrane thickness was approximately 300 µm. The prepared membrane pieces were affixed to the sensor probe using an adhesive solution prepared by dissolving 800 mg of PVC in 10 mL of THF.

### 2.3. Assembly of Electrodes and Surface Modification of the Lipid/Polymer Membrane

**Figure 3:**
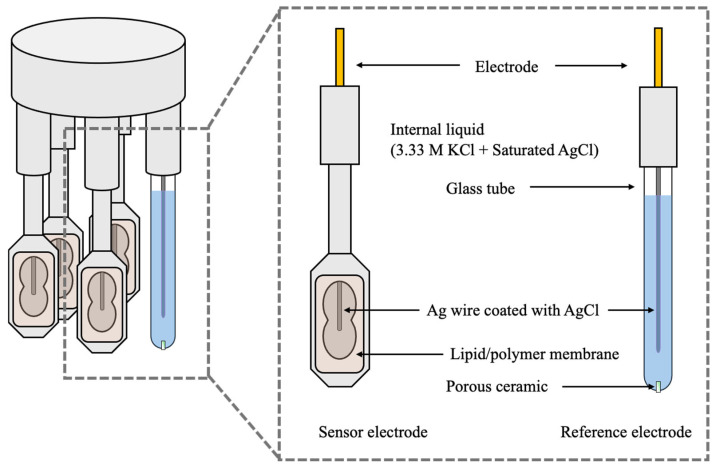
Schematic illustration of the configurations of the sensor and reference electrodes. Lipid/polymer membranes are attached to the sensor electrode.

The sensor probe with the attached lipid/polymer membrane was filled with an internal solution consisting of 3.33 M KCl and a saturated AgCl solution. An Ag/AgCl-coated silver wire was then inserted into the probe. The configurations of the sensor and reference electrodes, together with the taste sensing system, are shown in Figure 3. The reference electrode is set in the sample solution. In the simplest case, in this configuration, a negatively charged membrane interacts electrostatically with positively charged taste molecules, causing a positive potential change. A positive sensor response is also triggered when the membrane charge shifts in a positive direction, caused by the adsorption of taste substances onto the membrane or other related interactions [46].

Surface modification of the lipid/polymer membrane was carried out using the modifiers described above. Each modifier was dissolved in ultrapure water to prepare an aqueous solution with a concentration of 0.03 wt%. The assembled sensor electrodes were immersed in the modifier solutions for 72 h to modify the membrane surface. Measurements were also conducted using an unmodified lipid/polymer membrane (i.e., TDAB membrane) for comparison.

**Figure 4:**
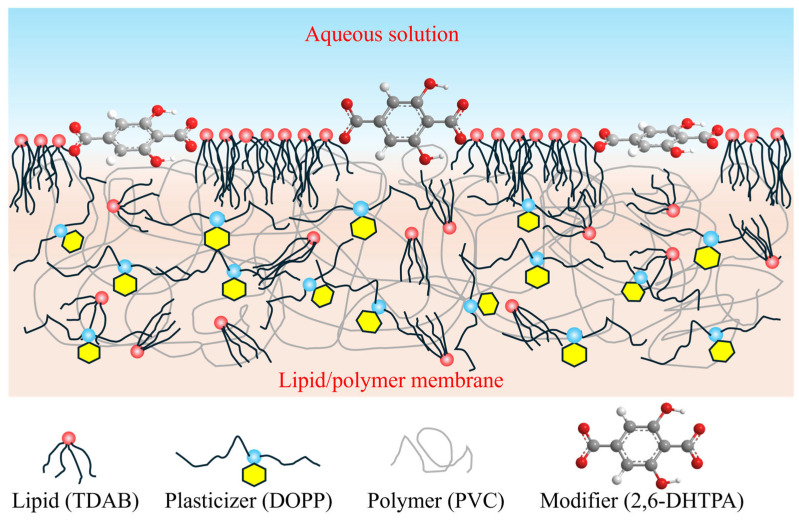
The state of 2,6-DHTPA in the lipid/polymer membrane.

Figure 4 illustrates the state of 2,6-DHTPA in the lipid/polymer membrane. The key point is that the hydrophobic moiety of the modifier remains within the hydrophobic interior of the membrane, while the functional groups capable of interacting with taste substances are oriented toward the solution. The modifier must possess a certain degree of hydrophobicity; if it is too hydrophilic, it cannot adsorb onto the lipid membrane, and hence the modification is unsuccessful. Based on this fact, the state of the modifier within the lipid membrane is considered to be determined by macroscopic factors such as hydrophilicity and hydrophobicity. Such a modification process—here referred to as preconditioning—has also been shown to alter the chemical composition of the membrane surface [47].

The reference solution used for taste measurements consisted of 30 mM KCl and 0.3 mM tartaric acid and was employed to determine the baseline potential. Sample solutions were prepared by dissolving MSG or IMP in the reference solution.

### 2.4. Measurement of Umami Substances Using a Taste Sensor

**Figure 5:**
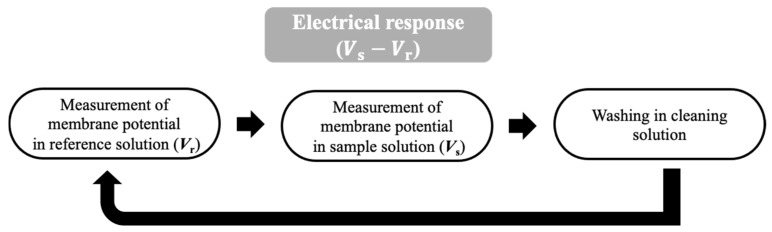
Measurement procedure of the taste sensor.

Taste measurements were performed using a TS-5000Z taste sensing system (Intelligent Sensor Technology, Inc., Kanagawa, Japan). The measurement procedure followed that described in our previous studies [42,45], as shown in Figure 5. The detection of umami substances using the taste sensor involved several steps. First, the sensor and reference electrodes (Figure 3) were immersed in the reference solution for 30 s, and the reference potential (*V*_r_) was recorded. Subsequently, the electrodes were immersed in the sample solution containing umami substances, and the sample potential (*V*_s_) was measured for 30 s. The sensor response was defined as the potential difference between *V*_r_ and *V*_s_. After measurement, the electrodes were immersed in a cleaning solution (10 mM KOH, 100 mM KCl, 30 vol% EtOH) to restore the initial charge state of the membrane surface, enabling subsequent measurements. This measurement cycle was repeated five times.

### 2.5. Statistical Analysis

For data analysis, the average of the third to fifth measurements from the five cycles using taste sensors, which exhibited stable responses, was used. For one measurement of MSG using a TPA-modified membrane, results from the second, third, and fifth cycles were used. The standard deviation (SD) of the data was calculated using *n* = 3 (electrodes) × 3 (rotations) = 9 values.

### 2.6. ^1^H-NMR Measurement

^1^H-NMR spectroscopy was used to analyze the molecular structures by observing the nuclear magnetic resonance of hydrogen nuclei. The ^1^H-NMR spectra were recorded using an ECS-400 spectrometer (JEOL, Tokyo, Japan). Deuterium water (D_2_O, 99.8 atom% D; Thermo Scientific Co., Tokyo, Japan) was used for the preparation of the sample solution. To reproduce the ionic conditions used in the taste sensor measurements, 1 mM KCl was added to the D_2_O sample solution. ^1^H-NMR spectra were referenced using 3-trimethylsilyl-1-propanesulfonic acid-d_6_ (DSS-d_6_, 98.0 atom% D; Santa Cruz Biotechnology Inc., Dallas, TX, USA) at 0.00 ppm. To prevent any unexpected interaction of compounds in the sample solution with DSS-d_6_, DSS-d_6_ dissolved in D_2_O was packed into a 3.5 mm stem coaxial inert NMR sample tube (Nihon Seimitsu Scientific Co., Tokyo, Japan), and the inert tube was repeatedly used for all samples by inserting it into a 5 mm NMR sample tube (Nihonseimitsu Scientific Co., Tokyo, Japan). The measurements at 25 °C were conducted under the following conditions: an acquisition time of 2.73 s, 8 scans, a relaxation delay of 12 s, automatic gain control, and a spinning rate of 15 Hz. The chemical shift is highly sensitive to structural changes and can be measured with great accuracy; therefore, almost any genuine binding interaction will result in a detectable shift [48,49].

## 3. Results and Discussion

### 3.1. Taste Sensor Responses

**Figure 6:**
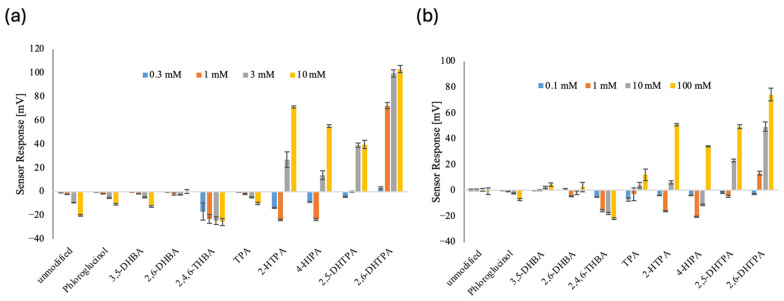
Electrical responses of the taste sensor to IMP (**a**) and MSG (**b**) for an unmodified membrane (i.e., a TDAB lipid membrane) and membranes modified with nine different modifiers. The IMP concentrations tested were 0.3, 1, 3, and 10 mM, while the MSG concentrations were 0.1, 1, 10, and 100 mM. The error bars indicate the SD of the data, *n* = 3 (electrodes) × 3 (rotations) = 9 values.

Figure 6 shows the taste sensor responses to IMP (a) and MSG (b) for an unmodified membrane (i.e., a TDAB lipid membrane) and membranes modified with nine different modifiers. The IMP concentrations tested were 0.3, 1, 3, and 10 mM, while the MSG concentrations were 0.1, 1, 10, and 100 mM.

Taste sensors employing membranes modified with four modifiers—namely, 2-HTPA, 4-HIPA, 2,5-DHTPA, and 2,6-DHTPA—exhibited clear positive potential responses to both IMP and MSG. The responses of 2,5- and 2,6-DHTPA modified membranes to IMP saturated at concentrations of several millimolar, with response magnitudes of approximately 40 mV and 100 mV, respectively. In contrast, all four modified membranes showed gradual potential responses to MSG over a concentration range from 1 to 100 mM.

Membranes modified with 2-HTPA and 4-HIPA exhibited transient negative responses at low concentrations of IMP and MSG. This behavior can be attributed to the fact that the pH of the reference solution was approximately 3.5, whereas the pH values of 0.3 mM IMP and 0.1 mM MSG solutions were around 4.0, and those of 1 mM IMP and MSG were approximately 6.2 and 4.5, respectively. This alkalization promoted the dissociation of the carboxyl groups of the modifiers, which had been limited in the reference solution. At higher concentrations of IMP and MSG, however, positive responses were observed due to interactions of IMP and MSG with the modifiers, as described below.

In fact, the carboxyl groups of 2-HTPA and 4-HIPA are not extensively dissociated in the reference solution, whereas those of 2,5- and 2,6-DHTPA, which exhibit almost no negative responses, are considerably dissociated. This can be inferred from the reference potential (*V*_r_). The *V*_r_ of the unmodified TDAB membrane is approximately 110 mV; in contrast, the *V*_r_ values of the membranes modified with 2-HTPA and 4-HIPA are approximately 80 mV and 70 mV, respectively, while those of the membranes modified with 2,5- and 2,6-DHTPA are about 50 mV. For the latter two modified membranes, dissociation of the carboxyl groups markedly reduces the positive charge from the original 110 mV state of the TDAB membrane, resulting in smaller *V*_r_ values. The acid dissociation constants (pKa) also support this conclusion. One of the pKa values of the four modifiers listed above is, in order, 2.3, 2.5, 1.8, and 1.2 (calculated from Marvin 23.7.0, ChemAxon, Budapest, Hungary), indicating that dissociation in the reference solution is not very advanced for the former two modifiers.

The remaining membranes exhibited either negative responses or responses close to zero over the entire concentration range of IMP and MSG. Although similar overall trends were observed for IMP and MSG in these cases, the magnitude of the negative response was larger for IMP than for MSG. This difference can be attributed to the fact that, near neutral pH, MSG carries an approximate charge of −1, whereas IMP carries a charge slightly less negative than −2 (i.e., a charge > −2). Differences and changes in the response potentials arising from variations in membrane charge will be discussed in detail later.

### 3.2. ^1^H-NMR Analysis

**Figure 7:**
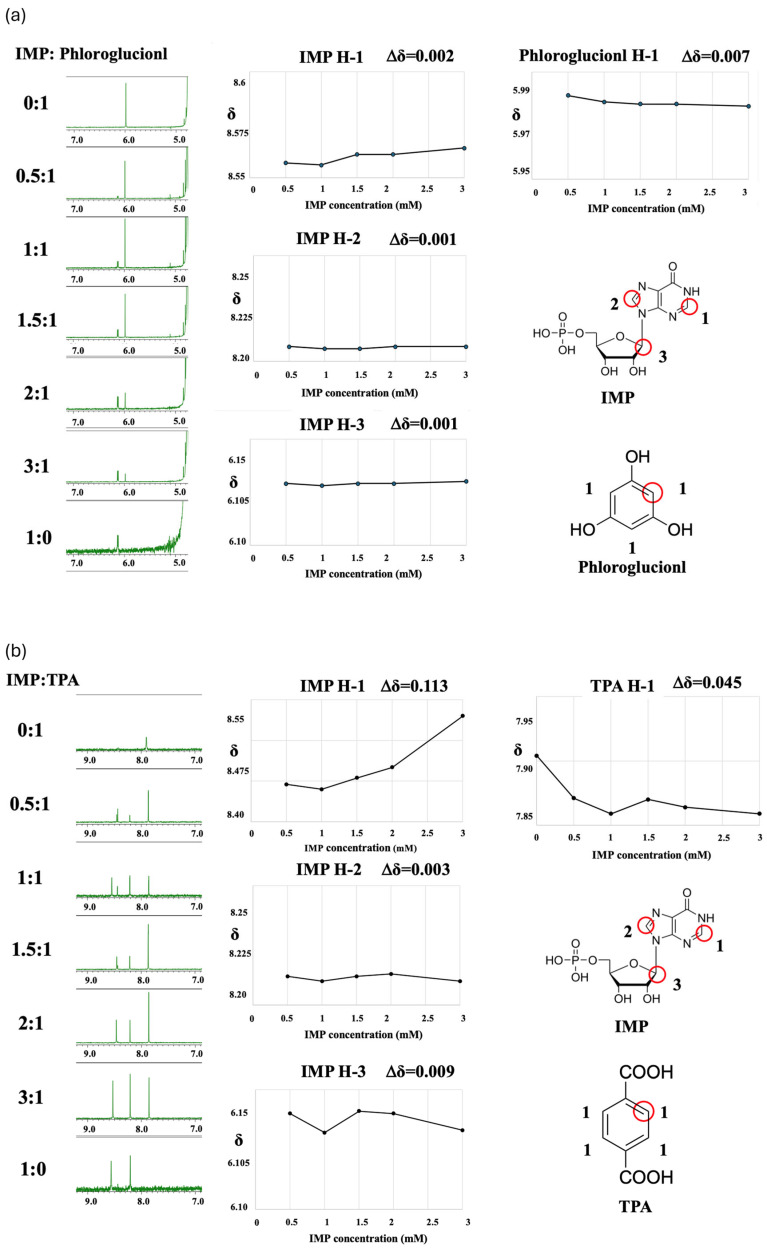

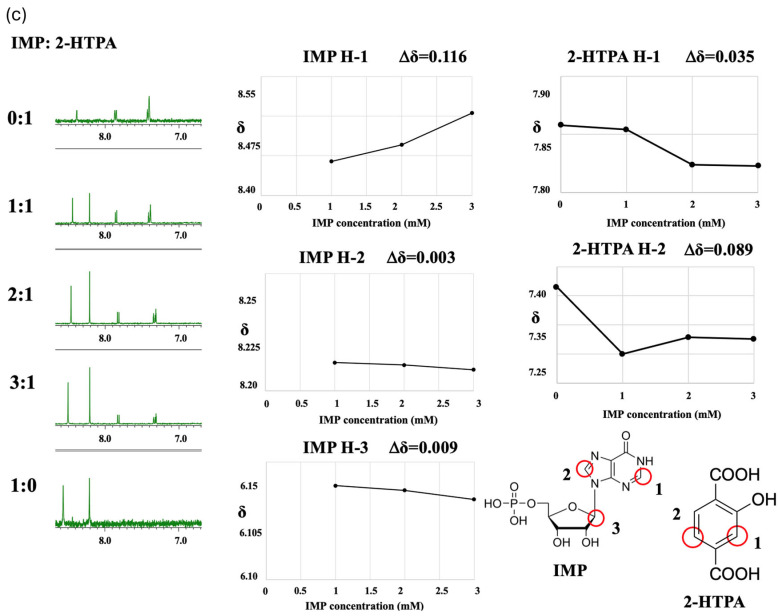
^1^H-NMR spectra and observed chemical shift changes for each modifier and IMP: (**a**) phloroglucinol, (**b**) TPA, and (**c**) 2-HTPA. Red circles indicate hydrogen atoms exhibiting noticeable chemical shift variations.

Figure 7 shows the chemical shift changes observed by ^1^H-NMR analysis for the modifiers phloroglucinol (a), TPA (b), and 2-HTPA (c) as a function of increasing IMP concentration. No significant chemical shift changes were observed for phloroglucinol, whereas pronounced chemical shift changes were observed for TPA and 2-HTPA.

With increasing molar ratio, the membrane modifier TPA (b) exhibited a clear change in its chemical resonance, shifting from 7.904 to 7.859, corresponding to a chemical shift change of 0.045 ppm. In the case of IMP, pronounced chemical shift changes were also observed for the hydrogen atoms at the 1- and 3-positions, with shift changes of 0.113 ppm and 0.009 ppm, respectively.

The chemical shift changes of 2-HTPA (c) were very similar to those observed for TPA. With increasing molar ratio, the membrane modifier 2-HTPA exhibited clear chemical shift changes of 0.035 ppm and 0.089 ppm at the 1- and 2-positions, respectively. Similarly, for IMP, substantial chemical shift changes of 0.116 ppm and 0.009 ppm were observed for the protons at the 1- and 3-positions, respectively.

These results indicate that IMP does not interact with phloroglucinol, whereas IMP does interact with TPA and 2-HTPA. The chemical shift changes in these modifiers and IMP over the full spectral range are shown in Figures S1–S3.

### 3.3. Relationship Between Potential Responses of Lipid Membranes with Various Modifiers and Chemical Shift Changes

Table 1 summarizes the responses of taste sensors equipped with unmodified lipid/polymer membranes (TDAB membrane) and membranes modified with nine different modifiers to 10 mM IMP, together with the chemical shift changes observed in the presence of both the modifiers and IMP. The modifiers are classified according to the number of carboxyl groups and the presence or absence of intramolecular hydrogen bonding.

From these results, it is evident that for four modifiers (2-HTPA, 4-HIPA, 2,5-DHTPA, and 2,6-DHTPA), chemical shift changes are observed, and the taste sensors exhibit considerable positive potential responses. The behaviors of both the taste sensor response and the chemical shift change for 2-HTPA are similar to those for 2,6-DHTPA [45]. For 3,5-DHBA, 2,4,6-THBA, and TPA, chemical shift changes are observed; however, the sensors exhibit either zero or negative potential responses. In contrast, for phloroglucinol and 2,6-DHBA, neither chemical shift changes nor potential responses are observed.

The unmodified membrane exhibits a negative potential response, which originates from electrostatic interactions between the positively charged lipid TDAB and IMP. Because the unmodified TDAB membrane is positively charged, it always interacts with negatively charged taste substances solely through electrostatic interactions and therefore lacks taste selectivity. Consequently, a negative potential response is undesirable for an umami sensor, whereas a positive potential response is preferable.

Table 1 suggests that receptor membranes without positive responses use modifiers lacking two carboxyl groups or intramolecular hydrogen bonding. Conversely, modifiers used in receptor membranes that exhibit positive responses contain two carboxyl groups and possess intramolecular hydrogen bonding.

For taste sensors employing modifiers that possess both intramolecular hydrogen bonding and two carboxyl groups, the following process occurs during IMP measurement. First, in these modifiers, the two carboxyl groups are dissociated due to intramolecular hydrogen bonding in the reference solution. Upon exposure to moderately high concentrations of IMP at 3 mM or above, intermolecular hydrogen bonds are formed between IMP and the modifier, causing the dissociated protons to return to the carboxyl groups and rendering the modifier electrically neutral. Because IMP carries a charge slightly less negative than −2 (i.e., a charge > −2) and the positive charge of the lipid TDAB remains unchanged, the surface charge of the membrane shifts from its initial value of −2 to a value of >−2. This change represents a net shift toward a positive charge. In other words, the transition from intramolecular to intermolecular hydrogen bonding induces a positive shift in membrane charge, resulting in a positive potential response of the taste sensor.

IMP and MSG solutions become more neutral in pH at higher concentrations from the acidic in the reference solution. As a result, the modifiers more readily dissociate protons. As shown in Figure 6, membranes modified with 2-HTPA, 4-HIPA, 2,5-DHTPA, and 2,6-DHTPA exhibit large positive responses to IMP. The dissociation constants of the carboxyl groups adjacent to the hydroxyl groups in these modifier molecules are 2.3, 2.5, 1.8, and 1.2 (calculated from Marvin 23.7.0, ChemAxon, Budapest, Hungary), respectively. For example, because the pH of a 10 mM IMP solution is 7.5, protons must dissociate from the carboxyl groups at such an IMP concentration. The possible potential response in that case would be in the negative direction. It suggests that the potential response is not caused by the pH change induced by IMP. Indeed, in the membrane modified with 2,6-DHBA for non-charged bitter substances, which utilizes the transition from intramolecular to intermolecular hydrogen bonding, almost the same positive response to caffeine is observed from acidic to mildly alkaline conditions; however, under strongly alkaline conditions, such as pH 12, the response disappears [50]. Conversely, as shown in Figure 6, the experimental results exhibit positive responses. This can only be interpreted as follows: the formation of intermolecular hydrogen bonds between these umami substances and the modifiers disrupts the intramolecular hydrogen bonds within the modifier molecules, causing the protons that had dissociated into the solution to return to the carboxyl groups of the modifiers. In fact, the NMR results support this conclusion.

Since the TPA molecule lacks a hydroxyl group and cannot form intramolecular hydrogen bonds, its modified membrane cannot exhibit a positive response even when interacting with IMP. In contrast, when a modifier such as 2,4,6-THBA possesses multiple hydroxyl groups and one adjacent carboxyl group that allows intramolecular hydrogen bonding, IMP can form hydrogen bonds with both the carboxyl and hydroxyl groups. However, because the charge shift from the initial value of −1 to a charge > −2 occurs toward the negative direction, the resulting potential response becomes negative. Although 3,5-DHBA also contains multiple hydroxyl groups and one carboxyl group, it does not possess intramolecular hydrogen bonding, and therefore, a positive potential response cannot be generated. In the case of 2,6-DHBA, which showed no chemical shift change, intramolecular hydrogen bonding is present; however, the molecule does not adopt a geometry that allows significant interaction with IMP, because the two hydroxyl groups are located adjacent to the carboxyl group. In other words, the carboxyl and hydroxyl groups are excessively localized on one side of the molecule, hindering their stable interaction with IMP.

For the unmodified membrane and the phloroglucinol-modified membrane, neither carboxyl groups nor hydroxyl groups are present. As a result, neither intramolecular hydrogen bonding nor intermolecular hydrogen bonding with IMP can occur, and only negative potential responses arising from simple electrostatic interactions are observed.

### 3.4. Interaction Mode Between IMP and the Modifier 2-HTPA

Here, for comparison, we turn our attention to the detection of electrically neutral bitter substances such as caffeine and theobromine using 2,6-DHBA, which has been shown to be effective for this purpose [50,51]. 2,6-DHBA possesses one carboxyl group flanked by two hydroxyl groups and therefore naturally forms intramolecular hydrogen bonding, resulting in a net charge of −1. When intermolecular hydrogen bonding and stacking (π–π interactions) occur between 2,6-DHBA and electrically neutral bitter substances, the intramolecular hydrogen bonding is disrupted, and the dissociated proton returns to the carboxyl group. Consequently, the charge of the complex formed between the modifier and the neutral bitter substance becomes zero, leading to a positive potential response of the sensor [50]. Although caffeine also interacts with 3,5-DHBA and resorcinol, no potential response was observed because these modifiers do not possess intramolecular hydrogen bonding [51]. This situation is analogous to the responses observed for 3,5-DHBA and TPA without intramolecular hydrogen bonding in the present study for IMP. The resulting responses in these cases are slightly negative because IMP carries a negative charge.

**Figure 8:**
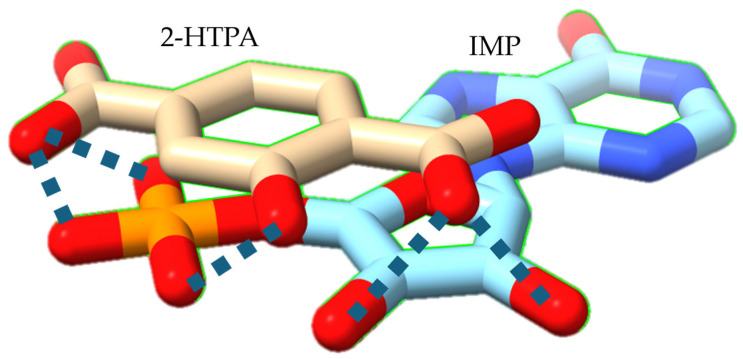
Proposed interaction model between IMP and 2-HTPA illustrated using UCSF ChimeraX 1.11.1 [52]. Blue dashed lines denote intermolecular hydrogen bonds.

Figure 8 illustrates the three-dimensional interaction between IMP and 2-HTPA. Because the three oxygen atoms of the phosphate group are equivalent, the charge > −2 and the bond character are evenly distributed across them, rather than being fixed as distinct single or double bonds. IMP forms intermolecular hydrogen bonds with both the carboxyl group and the hydroxyl group of 2-HTPA. That is, the initial intramolecular hydrogen bonding of the modifier 2-HTPA is transformed into intermolecular hydrogen bonding with IMP, causing the membrane charge to shift in the positive direction. As a result, the taste sensor employing the modified membrane exhibits a positive potential response to IMP.

In biological systems, the umami receptor T1R1/T1R3 recognizes the carboxyl group of MSG and the phosphate group of IMP [10,11,12]. Chemical reception and recognition by receptors arise from interactions at multiple sites, that is, multipoint interactions. Although the modifiers used in this study, such as 2-HTPA and 2,6-DHTPA, are small molecules, they interact with the carboxyl group of MSG and the phosphate group of IMP, thereby partially reproducing the function of umami receptors. Furthermore, taste sensor receptor membranes in which these modifiers are incorporated into lipid/polymer membranes can be regarded as highly simple functional devices that simultaneously perform two functions: chemical reception and conversion into an electrical potential. Based on the present findings, the construction of larger molecules may further approach the reception and recognition functions observed in biological systems.

In this study, the pH was not adjusted during the measurement of umami substances; instead, it was allowed to fluctuate depending on the IMP or MSG concentration. The intensity of umami perceived by humans is pH-dependent [1,2,3,4,12]. For example, in acidic regions such as pH 3 or 4, the carboxyl groups of the umami substance MSG are no longer in a dissociated state. This weakens their binding to umami receptors, which simply leads to a decrease in umami intensity. Therefore, the issue of pH is not straightforward. To practicalize the results of this research and development in the future, it will be necessary to perform measurements not only on actual food samples but also under various conditions with varying pH levels, coexisting ion species, and surrounding environments.

## 4. Conclusions

In this study, we systematically investigated the structural requirements of membrane modifiers necessary for the potentiometric detection of IMP using taste sensors based on lipid/polymer membranes. By employing nine modifiers with different chemical structures and combining taste sensor measurements with ^1^H-NMR analysis, three distinct response patterns were identified: modifiers that induced both chemical shift changes and positive potential responses, modifiers that exhibited chemical shift changes but yielded nearly zero or negative potential responses, and modifiers that showed neither chemical shift changes nor potential responses.

Detailed comparison of these patterns revealed that modifiers failing to produce positive potential responses lacked either two carboxyl groups or intramolecular hydrogen bonding involving hydroxyl groups. These findings clearly demonstrate that the presence of both (i) two carboxyl groups and (ii) intramolecular hydrogen bonding within the modifier molecule constitutes the essential structural requirements for generating a positive potential response to IMP.

The results further suggest that the positive sensor response arises from a transition from intramolecular hydrogen bonding within the modifier to intermolecular hydrogen bonding between the modifier and IMP, accompanied by a redistribution of charge at the membrane surface. This mechanism is consistent with the observed chemical shift changes in both the modifier and IMP, as well as with the potentiometric behavior of the sensor.

The present study provides molecular-level insight into the interaction mechanism between IMP and membrane modifiers, thereby advancing the understanding of umami sensing using taste sensors. The taste sensor with modified membranes can evaluate the synergistic effect occurring in the coexistence of IMP and MSG [45]. These findings are expected to contribute to the rational design of membrane modifiers for selective umami detection and to the further development of taste sensors capable of quantitatively evaluating umami substances and their synergistic effects.

## Figures and Tables

**Table 1:** Responses of the taste sensor incorporating nine different modifiers, as well as without modifiers, to 10 mM IMP, together with the corresponding chemical shift changes. The results are classified according to the number of carboxyl groups and the presence of intramolecular hydrogen bonding.

Carboxyl Groups	0		1			2				
Intramolecular H-Bond	No	No	No	Yes	Yes	No	Yes	Yes	Yes	Yes
Modifier	Non	Phloroglucinol	3,5-DHBA	2,6-DHBA	2,4,6-THBA	TPA	2-HTPA	4-HIPA	2,5-DHTPA	2,6-DHTPA
Taste sensor response (mV)	−20	−11	−13	−5	−26	−10	71	55	40	103
Chemical shift change	-	No	Yes	No	Yes	Yes	Yes	Yes	Yes	Yes

## Data Availability

The original contributions presented in this study are included in the article/Supplementary Material. Further inquiries can be directed to the corresponding author.

## Supplementary Materials

The following supporting information can be downloaded at: https://www.mdpi.com/article/10.3390/s26061787/s1, Figures S1, S2 and S3: Full ^1^H-NMR spectra of mixtures of IMP with phloroglucinol, TPA, and 2-HTPA, respectively.

## References

[1] Ikeda K. (1909). On a new seasoning. J. Tokyo Chem. Soc..

[2] Lindemann B., Ogiwara Y., Ninomiya Y. (2002). The discovery of umami. Chem. Senses.

[3] Cairoli P., Pieraccini S., Sironi M., Morelli C.F., Speranza G., Manitto P. (2008). Studies on umami taste: Synthesis of new guanosine 5′-phosphate derivatives and their synergistic effect with monosodium glutamate. J. Agric. Food Chem..

[4] Nakamura E. (2011). One hundred years since the discovery of the “umami” taste from seaweed broth by Kikunae Ikeda, who transcended his time. Chem. Asian J..

[5] Yamaguchi S., Ninomiya K. (2000). Umami and food palatability. J. Nutr..

[6] Wijayasekara K., Wansapala J. (2017). Uses, effects and properties of monosodium glutamate (MSG) on food & nutrition. Int. J. Food Sci. Nutr..

[7] Morita R., Ohta M., Hayabuchi H., Fujitani S., Yoshida S., Matsumoto H., Tsuchihashi T. (2020). Quantitative verification of the effect of using an umami substance (L-glutamate) to reduce salt intake. Hypertens. Res..

[8] Kumazawa T., Kurihara K. (1990). Large synergism between monosodium glutamate and 5′-nucleotides in canine taste nerve responses. Am. J. Physiol..

[9] Rotola-Pukkila M., Yang B., Hopia A. (2019). The effect of cooking on umami compounds in wild and cultivated mushrooms. Food Chem..

[10] Mouritsen O.G., Khandelia H. (2012). Molecular mechanism of the allosteric enhancement of the umami taste sensation. FEBS J..

[11] Zhang F., Klebansky B., Fine R.M., Xu H., Pronin A., Liu H., Tachdjian C., Li X. (2008). Molecular mechanism for the umami taste synergism. Proc. Natl. Acad. Sci. USA.

[12] Liu H., Da L.-T., Liu Y. (2019). Understanding the molecular mechanism of umami recognition by T1R1–T1R3 using molecular dynamics simulations. Biochem. Biophys. Res. Commun..

[13] San Gabriel A., Maekawa T., Uneyama H., Yoshie S., Torii K. (2007). mGluR1 in the fundic glands of rat stomach. FEBS Lett..

[14] Escuder-Gilabert L., Peris M. (2010). Highlights in recent applications of electronic tongues in food analysis. Anal. Chim. Acta.

[15] Banerjee R., Tudu B., Bandyopadhyay R., Bhattacharyya N. (2016). A review on combined odor and taste sensor systems. J. Food Eng..

[16] Tan J., Xu J. (2020). Applications of electronic nose (e-nose) and electronic tongue (e-tongue) in food quality-related properties determination: A review. Artif. Intell. Agric..

[17] Uchida T. (2024). Taste sensor assessment of bitterness in medicines: Overview and recent topics. Sensors.

[18] Rudnitskaya A., Kirsanov D., Blinova Y., Legin E., Seleznev B., Clapham D., Ives R.S., Saunders K.A., Legin A. (2013). Assessment of bitter taste of pharmaceuticals with multisensor system employing three-way PLS regression. Anal. Chim. Acta.

[19] Shimizu F.M., de Barros A., Braunger M., Gaál G., Riul A. (2023). Information visualization and machine learning driven methods for impedimetric biosensing. TrAC Trends Anal. Chem..

[20] Mohamed-Ahmed A.H.A., Soto J., Ernest T., Tuleu C. (2016). Non-human tools for the evaluation of bitter taste in the design and development of medicines: A systematic review. Drug Discov. Today.

[21] Toko K. (2023). Research and development of taste sensors as a novel analytical tool. Proc. Jpn. Acad. Ser. B.

[22] Hayashi N., Ujihara T., Hayakawa F., Nakano Y., Kawakami T., Ikezaki H. (2020). Standardization of tomato juice taste using a taste sensor approach. Biosci. Biotechnol. Biochem..

[23] Hou F., Fan X., Gui X., Li H., Li H., Wang Y., Shi J., Zhang L., Yao J., Li X. (2023). Development of a variety and quality evaluation method for Amomi fructus using GC, electronic tongue, and electronic nose. Front. Chem..

[24] Zhang H., Zou G., Liu X., Xiao Y., Wang W. (2019). Identification of Xinyang Maojian tea taste using electronic tongue. Sens. Mater..

[25] Ujihara T., Hayashi N., Ikezaki H. (2013). Objective evaluation of astringent and umami taste intensities of Matcha using a taste sensor system. Food Sci. Technol. Res..

[26] Pardo H., Owoyemi A., Benjamin O., Goldenberg L., Yaniv Y., Doron-Faigenboim A., Carmi N., Porat R. (2021). Sensory analysis of a new citrus juice made from ‘Aliza’ fruit: A new pomelo × mandarin hybrid. J. Food Sci. Nutr. Res..

[27] Makita Y., Ishida T., Kobayashi N., Fujio M., Fujimoto K., Moritomo R., Fujita J., Fujiwara S. (2016). Evaluation of the bitterness-masking effect of powdered roasted soybeans. Foods.

[28] Riul A., Correa D.S., Shimizu F.M., Braunger M.L., Riul A. (2021). A first taste to electronic tongues. Electronic Tongues: Fundamentals and Recent Advances.

[29] Shimizu F.M., Gaál G., Braunger M.L., Riul A., Shimizu F.M., Braunger M.L., Riul A. (2021). Recent developments on devices applied to impedimetric electronic tongues. Electronic Tongues: Fundamentals and Recent Advances.

[30] Cetó X., Del Valle M. (2022). Electronic tongue applications for wastewater and soil analysis. iScience.

[31] Wesoly M., Przewodowski W., Ciosek-Skibińska P. (2023). Electronic noses and electronic tongues for the agricultural purposes. TrAC Trends Anal. Chem..

[32] Lu L., Hu Z., Hu X., Li D., Tian S. (2022). Electronic tongue and electronic nose for food quality and safety. Food Res. Int..

[33] Zhao X., Qiu W., Shao X., Fu B., Qiao X., Yuan W., Yang M., Liu P., Du M., Tu M. (2024). Identification, screening and taste mechanisms analysis of two novel umami pentapeptides derived from the myosin heavy chain of Atlantic cod (*Gadus morhua*). RSC Adv..

[34] Podraźka M., Bączyńska E., Kundys M., Jeleń P.S., Nery E.W. (2018). Electronic tongue—A tool for all tastes?. Biosensors.

[35] Chen W., Huang Y., Jiang S., Chen G., Liu Y. (2020). Research on sensing characteristics of three human umami receptors via receptor-based biosensor. Flavour Fragr. J..

[36] Li M., Fan M., Fu B., Chang Z., Yi J., Xie Y., Ren W., Tian Y., Zhao Q., Cheng S. (2025). A novel strategy based on mouse organoid biosensor for detecting umami substances and their synergistic effect. Food Chem..

[37] Tian Y., Wang P., Du L., Wu C. (2022). Advances in gustatory biomimetic biosensing technologies: In vitro and in vivo bioelectronic tongue. TrAC Trends Anal. Chem..

[38] Du L., Chen W., Tian Y., Zhu P., Wu C., Wang P. (2020). A biomimetic taste biosensor based on bitter receptors synthesized and purified on chip from a cell-free expression system. Sens. Actuators B Chem..

[39] Cetó X., Céspedes F., Del Valle M. (2013). Assessment of individual polyphenol content in beer by means of a voltammetric bioelectronic tongue. Electroanalysis.

[40] Kong L., Dong Y., Shu G., Feng Y., Zhu M. (2024). Multienzyme-mediated dual-channel magnetic relaxation switching taste biosensor (D-MRSTB) for simultaneous detection of umami compounds and synergistic enhancement in food. ACS Sens..

[41] Iiyama S., Kuga H., Ezaki S., Hayashi K., Toko K. (2003). Peculiar change in membrane potential of taste sensor caused by umami substances. Sens. Actuators B Chem..

[42] Yuan W., Zhao Z., Kimura S., Toko K. (2024). Development of taste sensor with lipid/polymer membranes for detection of umami substances using surface modification. Biosensors.

[43] Yuan W., Ide H., Zhao Z., Koshi M., Kimura S., Matsui T., Toko K. (2024). Investigating the mechanism underlying umami substance detection in taste sensors by using 1H-NMR analysis. Chemosensors.

[44] Yuan W., Otsuka S., Jin J., Onodera T., Yatabe R., Kimura S., Toko K. (2025). Relationship between sensor sensitivity and chemical structure of benzene-carboxylic modifiers for umami substance detection. Chemosensors.

[45] Otsuka S., Koshi M., Onodera T., Yatabe T., Matsui T., Toko K. (2025). Detection of inosine monophosphate and the umami synergistic effect using a taste sensor with a surface-modified membrane. Molecules.

[46] Oohira K., Toko K. (1996). Theory of electric characteristics of the lipid/PVC/DOPP membrane and PVC/DOPP membrane in response to taste stimuli. Biophys. Chem..

[47] Yatabe R., Noda J., Tahara Y., Naito Y., Ikezaki H., Toko K. (2015). Analysis of a lipid/polymer membrane for bitterness sensing with a preconditioning process. Sensors.

[48] Williamson M.P. (2013). Using chemical shift perturbation to characterise ligand binding. Prog. Nucl. Magn. Reson. Spectrosc..

[49] Govindaraju V., Young K., Maudsley A.A. (2000). Proton NMR chemical shifts and coupling constants for brain metabolites. NMR Biomed..

[50] Yoshimatsu J., Toko K., Tahara Y., Ishida M., Habara M., Ikezaki H., Kojima H., Ikegami S., Yoshida M., Uchida T. (2020). Development of taste sensor to detect non-charged bitter substances. Sensors.

[51] Ishida M., Ide H., Arima K., Zhao Z., Matsui T., Toko K. (2022). Identification of the principle of taste sensors to detect non-charged bitter substances by 1H-NMR measurement. Sensors.

[52] Meng E.C., Goddard T.D., Pettersen E.F., Couch G.S., Pearson Z.J., Morris J.H., Ferrin T.E. (2023). UCSF ChimeraX: Tools for structure building and analysis. Protein Sci..

