# Supplementary material for: Effect of the Chemical Structure of Modifiers Used in the Receptive Membrane of an Umami Taste Sensor on Its Electrical Responses
Source: Sensors (Basel). 2026 Mar 12;26(6):1787. doi: 10.3390/s26061787 (PMC13029917; doi:10.3390/s26061787)
Supplement: Supplementary file 1 [file sensors-26-01787-s001.zip › sensors-4161693-supplementary.pdf]

Supplementary Materials

Article

Effect of the Chemical Structure of Modifiers Used in the Receptive Membrane of an Umami Taste Sensor on Its Electrical Responses

Kiyoshi Toko [1,2,3,4,*], Sota Otsuka [5], Mariko Koshi [6], Yuzuki Koga [6], Takeshi Onodera [7], Rui Yatabe [7] and Toshiro Matsui [3,6]

1 Food and Health Innovation Center, Nakamura Gakuen University, 5-7-1 Befu, Fukuoka 814-0198, Japan
2 Graduate School of Nutritional Sciences, Nakamura Gakuen University, 5-7-1 Befu, Fukuoka 814-0198, Japan
3 Research and Development Center for Five-Sense Devices, Kyushu University, 744 Motooka, Fukuoka 819-0395, Japan
4 Institute for Advanced Study, Kyushu University, 744 Motooka, Fukuoka 819-0395, Japan
5 Graduate School of Information Science and Electrical Engineering, Kyushu University, 744 Motooka, Fukuoka 819-0395, Japan
6 Department of Bioscience and Biotechnology, Faculty of Agriculture, Graduate School of Kyushu University, 744 Motooka, Nishi-ku, Fukuoka 819-0395, Japan
7 Faculty of Information Science and Electrical Engineering, Kyushu University, 744 Motooka, Fukuoka 819-0395, Japan
* Correspondence: ktoko@nakamura-u.ac.jp

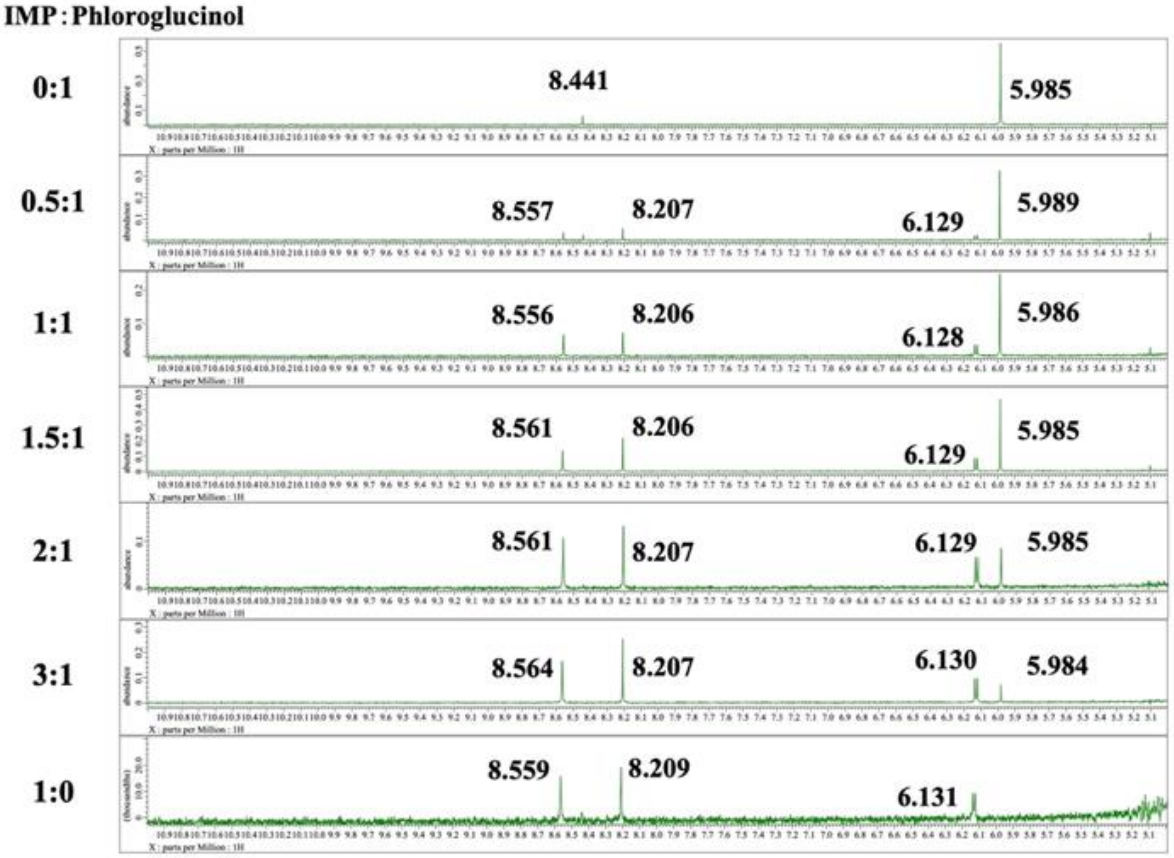

Figure S1. Full ^{1}H-NMR spectra of mixtures of IMP with phloroglucinol.

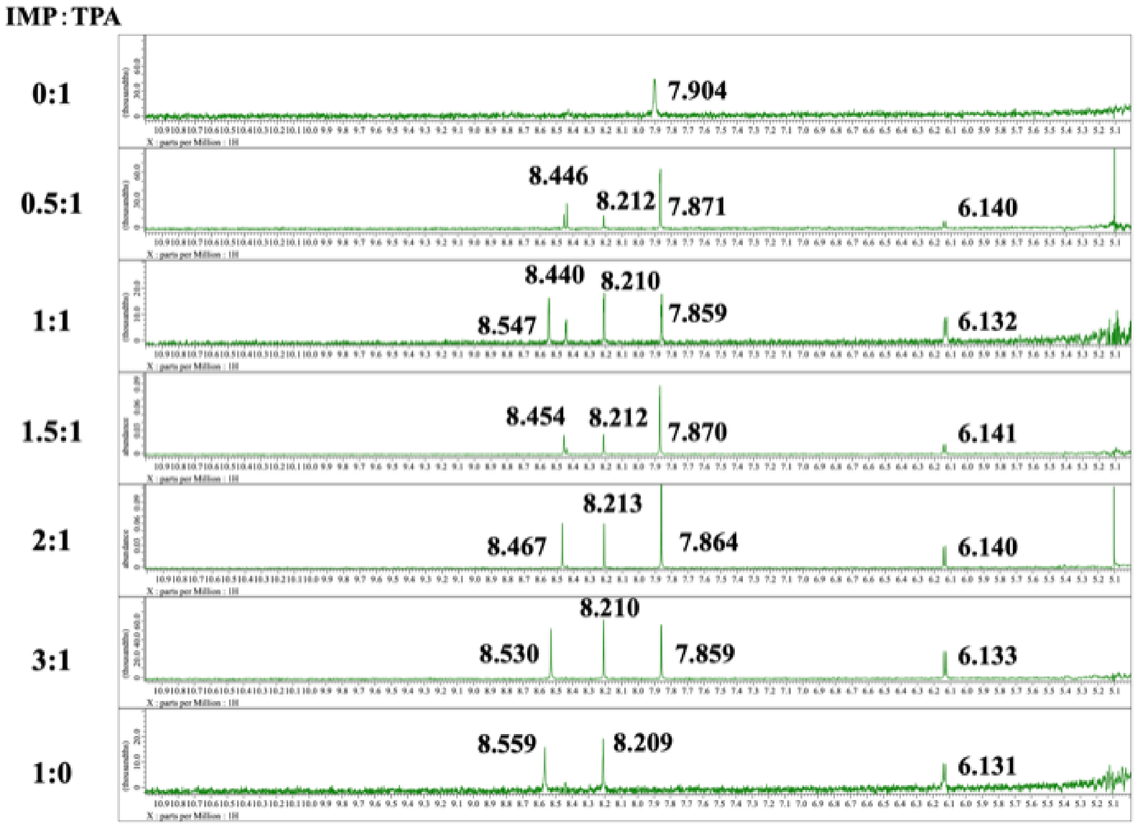

Figure S2. Full ¹H-NMR spectra of mixtures of IMP with TPA.

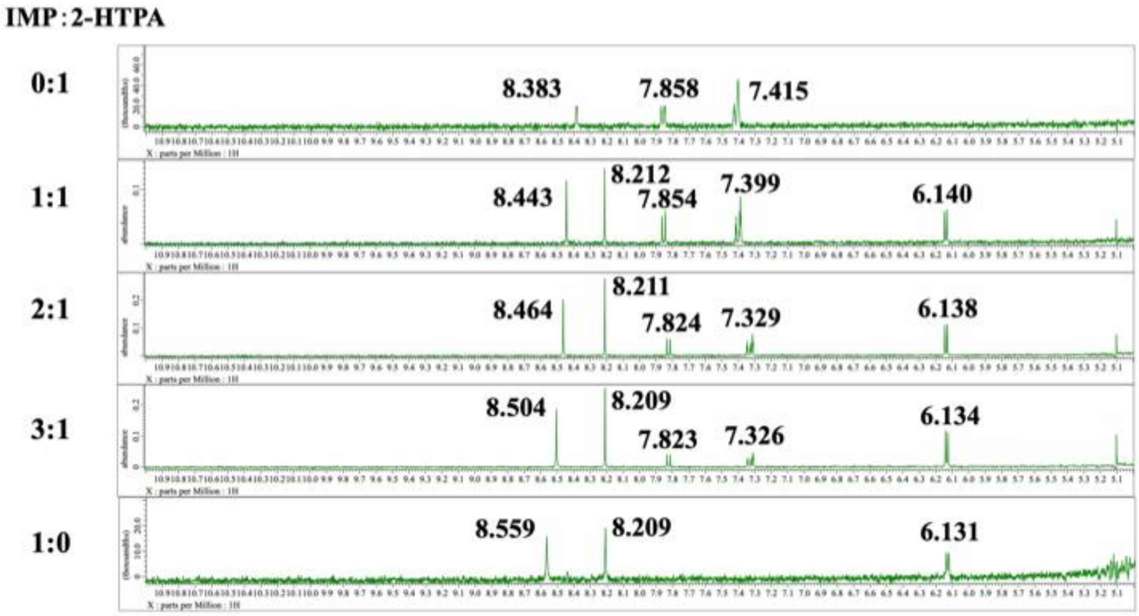

Figure S3. Full ¹H-NMR spectra of mixtures of IMP with 2-HTPA.
